# The NLPR3 inflammasome and obesity‐related kidney disease

**DOI:** 10.1111/jcmm.13333

**Published:** 2017-08-31

**Authors:** Ben Ke, Wen Shen, Xiangdong Fang, Qinghua Wu

**Affiliations:** ^1^ Department of Nephrology The Second Affiliated Hospital of Nanchang University Nanchang Jiangxi China; ^2^ Department of Cardiovascular Medicine The Second Affiliated Hospital of Nanchang University Nanchang Jiangxi China

**Keywords:** NLRP3 inflammation, obesity‐related kidney disease, mitochondrial dysfunction, endoplasmic reticulum stress, inflammation

## Abstract

Over the past decade, the prevalence of obesity has increased, accompanied by a parallel increase in the prevalence of chronic kidney disease (CKD). Mounting evidence suggests that high body mass index (BMI) and obesity are important risk factors for CKD, but little is known about the mechanisms of obesity‐related kidney disease (ORKD). The NLRP3 inflammasome is a polyprotein complex that plays a crucial role in the inflammatory process, and numerous recent studies suggest that the NLRP3 inflammasome is involved in ORKD development and may serve as a key modulator of ORKD. Moreover, inhibiting activation of the NLRP3 inflammasome has been shown to attenuate ORKD. In this review, we summarize recent progress in understanding the link between the NLRP3 inflammasome and ORKD and discuss targeting the NLRP3 inflammasome as a novel therapeutic approach for ORKD.

**Table:** 

**• Introduction**
**• Potential mechanisms of ORKD**
**•The NLRP3 inflammasome and the renin–angiotensin–aldosterone system**
**• The NLRP3 inflammasome and mitochondrial dysfunction**
**• The NLRP3 inflammasome and endoplasmic reticulum stress**
**• The NLRP3 inflammasome and inflammation**
**• Conclusion and perspective**
**• Acknowledgements**
**• Conflict of interests**

## Introduction

Obesity has become a serious public health problem worldwide. According to data from the US Centers for Disease Control and Prevention, only approximately 15% of the American population was obese in 1988, which is defined as having a BMI >30 kg/m 1. However, more than 25% of the adult population was overweight in 42 states, and more than 30% of the population was obese in 13 states in 2012 1. In 2014, more than 35% of American adults (78 million) and 17% of children (12.5 million) were obese 2. Currently, over 600 million adults (>18 years) are obese worldwide 3, and the increasing prevalence of obesity has significant implications for cardiovascular disease and CKD. Higher BMI is associated with the presence and development of proteinuria in individuals without kidney disease 4, 5, 6, 7. Furthermore, numerous large population‐based studies have shown that higher BMIs are associated with the presence and development of a low estimated glomerular filtration rate (GFR), rapid loss of estimated GFR over time 8 and end‐stage renal disease 9. Elevated BMI and obesity have been shown to be associated with rapid CKD progression in patients with pre‐existing CKD 10.

The NLRP3 inflammasome is an approximately 700 kD polyprotein complex that plays a crucial role in the inflammatory process 11. This inflammasome is associated with the apoptosis‐associated speck‐like protein that contains a caspase recruitment domain (ASC), which recruits caspase‐1 and induces its activation. Caspase‐1 is known as an IL‐1β‐converting enzyme to process pro‐IL‐1β into its mature IL‐1β form and induce its release, leading to inflammation and tissue damage 12. Inflammasomes are reportedly present in podocytes, mesangial cells and intercalated cells in human kidneys 12, 13 although some controversy regarding inflammasome location remains 14. Recently, numerous studies have shown the NLRP3 inflammasome to be involved in the onset of kidney disease, including diabetic nephropathy, IgA nephropathy, crystal‐induced nephropathy and ORKD 12, 15, 16. This review focuses on the role of the NLRP3 inflammasome in the development and progression of ORKD, which may provide a novel therapeutic strategy for ORKD treatment.

## Potential mechanisms of ORKD

**Figure 1:**
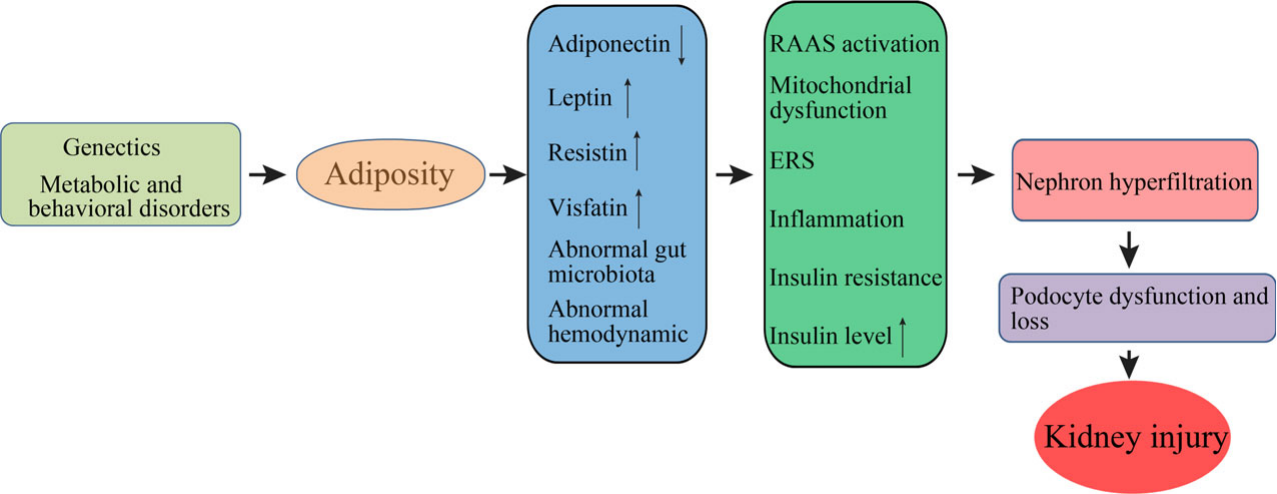
Adiposity leads to kidney injury. Schematic representation of the processes by which adiposity contributes to kidney injury.

Obesity has long been associated with kidney diseases 17, including glomerulopathies, nephrolithiasis and poor renal graft survival. ORKD is essentially single‐nephron hyperfiltration caused by the ratio of nephrons to body mass being reduced, and it has been recognized as a distinct entity characterized by glomerulomegaly, progressive glomerulosclerosis and renal functional decline 18. While the exact mechanisms by which obesity worsens or causes CKD remain unclear, adiposity is known to cause kidney injury (Fig. 1). Some deleterious renal consequences of obesity may be mediated by downstream co‐morbidity conditions, such as diabetes mellitus or hypertension, but adiposity has been shown to directly impact the kidneys by inducing the production of adiponectin 19, leptin 20 and resistin 21, and by disrupting gut microbiota 22. These components activate the renin–angiotensin–aldosterone system 23, mitochondrial dysfunction 24, endocytoplasmic reticulum stress 25, inflammation 2, and increase the production of insulin and insulin resistance 26. Thus far, obesity, especially morbid obesity, is believed to induce afferent vasodilation to augment the glomerular filtration rate, or more specifically, nephron hyperfiltration 27. If glomerular autoregulation is impaired, systemic blood pressure can be transmitted to the glomerular capillaries, leading to barotrauma. Glomerular capillary dilation may increase barotrauma susceptibility, which would require podocytes to cover a larger surface, thereby leading to proteinuria 27.

Overweight and obesity are associated with hemodynamics, which manifests as nephron hyperfiltration 28. He *et al*. found renal hemodynamic changes in early‐stage obesity‐related nephropathy in children 29, and Anastasio *et al*. found that a modified hemodynamic response to a protein meal may be the earliest hallmark of future kidney dysfunction in obese subjects 30. Moreover, obesity is a status of systemic inflammation, which leads to aberrant renal perfusion 31. In mice adipose tissue, the NLRP3 inflammasome was shown to promote high‐fat diet (HFD)‐induced inflammation 32, which may cause or worsen ORKD. Therefore, the NLRP3 inflammasome is associated with various cell types inside and outside the kidney in ORKD.

Diabetes mellitus, especially type 2 diabetes mellitus (T2DM), is a classic risk factor for ORKD 27, and the role of sodium‐glucose cotransporter‐2 (SGLT‐2) in diabetic nephrology has recently attracted attention. SGLT‐2, a member of the sodium‐glucose transporter family, regulates renal glucose reabsorption in the proximal tubule 33 and reportedly drives the deactivation of tubuloglomerular feedback and hyperglycaemia‐induced glomerular hyperfiltration 34, which is obviously aggravated by concomitant obesity. Furthermore, SGLT2 inhibitors increase the delivery of fluid and electrolytes to the macula densa, thereby activating tubuloglomerular feedback and increasing tubular back pressure, which mitigates glomerular hyperfiltration, reduces oxygen demand in the kidney and lessens albuminuria 34. Therefore, SGLT2 may play an active role in ORKD. Ye *et al*. found that SGLT‐2 inhibition with dapagliflozin reduced activation of the NLRP3/ASC inflammasome in T2DM mice 35. Unfortunately, literature on the relationship between SGLT‐2 and the NLRP3 inflammasome in ORKD is lacking.

## The NLRP3 inflammasome and the renin–angiotensin–aldosterone system

The renin–angiotensin–aldosterone system (RAAS), known for its role in regulating blood pressure, fluids and electrolyte balance, plays an active role in ORKD. In addition, the RAAS is involved in many physiological and pathological processes, including tissue growth; hypertrophy; inflammation; and glucose, lipid and energy metabolism 36. A RAAS blockade was shown to be highly effective in retarding the progression of renal disease in human and experimental animals 37, and increased RAAS activity, predominantly in visceral adipose tissue, is a characteristic of obesity 38. Moreover, expression of all the RAAS components, including angiotensinogen (AGT), angiotensin‐converting enzyme (ACE), renin, and the Ang II type 1 (AT1) and type 2 (AT2) receptors 39, has been detected in primary cultured adipocytes, human adipose tissue and commonly used rodent models. Furthermore, Ang II overexpression in adipose tissue and imbalance between the activated AT1 and AT2 receptor‐mediated effects were shown to contribute to ORKD 40. In addition, RAAS activation has been shown to promote the secretion of aldosterone, which leads to renal damage 41.

**Figure 2:**
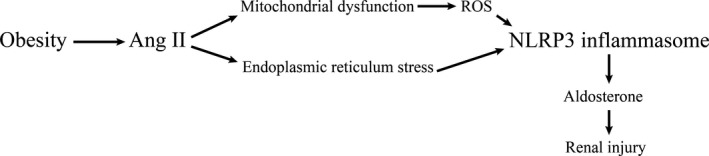
Obesity promotes the production of Ang II, which activates the NLRP3 inflammasome *via* mitochondrial dysfunction or ERS. The NLRP3 inflammasome then induces renal injury *via* aldosterone.

The RAAS may promote ORKD by activating the NLRP3 inflammasome (Fig. 2), as Wang *et al*. found that an RAS blockade using the renin inhibitor aliskiren prevented up‐regulation of the NLRP3 inflammasome component ASC and inhibited caspase‐1 activation and subsequent IL‐1β processing and release 42. Moreover, NLRP3 deletion was shown to protect tubular epithelial cells (TECs) from Ang II‐triggered mitochondrial dysfunction and NLRP3 inflammasome activation. Thus, Ang II induces NLRP3 inflammasome activation, which is mediated by mitochondrial dysfunction, in TECs 43. Furthermore, mitochondrial dysfunction was shown to cause the production of reactive oxygen species (ROS), which mediate NLRP3 inflammasome activation and contribute to aldosterone‐induced renal tubular cell injury 44. A recent study showed that Ang II could induce NLRP3 inflammasome activation through endoplasmic reticulum stress (ERS), which is a physiological or pathological condition caused by glucose deprivation, hypoxia, viral infection, or the Ang II‐induced overexpression of NLRP3, ASC, caspase‐1, IL‐1β and IL‐18 45. Eventually, NLRP3 inflammasome activation contributes to aldosterone‐induced renal injury 46.

Epithelial–mesenchymal transition (EMT) is a process through which the renal epithelial phenotype is transformed into a mesenchymal phenotype in response to cell damage, leading to (ECM) accumulation and myofibroblast production, which is the key effector in the synthesis and deposition of the ECM complex 47. On one hand, NLRP3 activation in TECs was shown to drive the EMT during progressive renal fibrosis 48, as Nlrp3^−/−^ mice displayed reduced tubular injury and interstitial fibrosis upon unilateral ureteral ligation compared with wild‐type animals 48. On the other hand, Ang II was shown to induce hepatocyte EMT by promoting the NOX‐derived, H_2_O_2_^−^activated NLRP3 inflammasome/IL‐1β/Smad pathway 49. Although Ang II induces NLRP3 inflammasome activation, little is known about the impact of the NLRP3 inflammasome on the RAAS.

## The NLRP3 inflammasome and mitochondrial dysfunction

Mitochondria participate in numerous cellular functions, including ion homeostasis, heme and steroid synthesis, calcium signalling and apoptosis 50. The prominent role of this organelle is to generate energy for cellular metabolism using the oxidative phosphorylation system. Emerging evidence suggests that dysfunctional mitochondria play a primary role in the development of CKD and co‐morbidities related to CKD 50. Mitochondrial dysfunction is reportedly involved in obesity and obesity‐related disorders 24, 51 and can initiate autophagy, a cellular degradation pathway essential for survival, to remove dysfunctional mitochondria and maintain cellular homeostasis 52. Yamamoto *et al*. found that autophagy ablation exaggerated HFD‐induced mitochondrial dysfunction and inflammasome activation and proposed that HFD‐impaired autophagic flux contributes to kidney lipotoxicity 53. Moreover, protected mitochondria have been shown to overcome lipotoxicity in the kidney 25. Considering these studies, a close relationship between mitochondrial dysfunction and ORKD was determined to exist 54.

**Figure 3:**
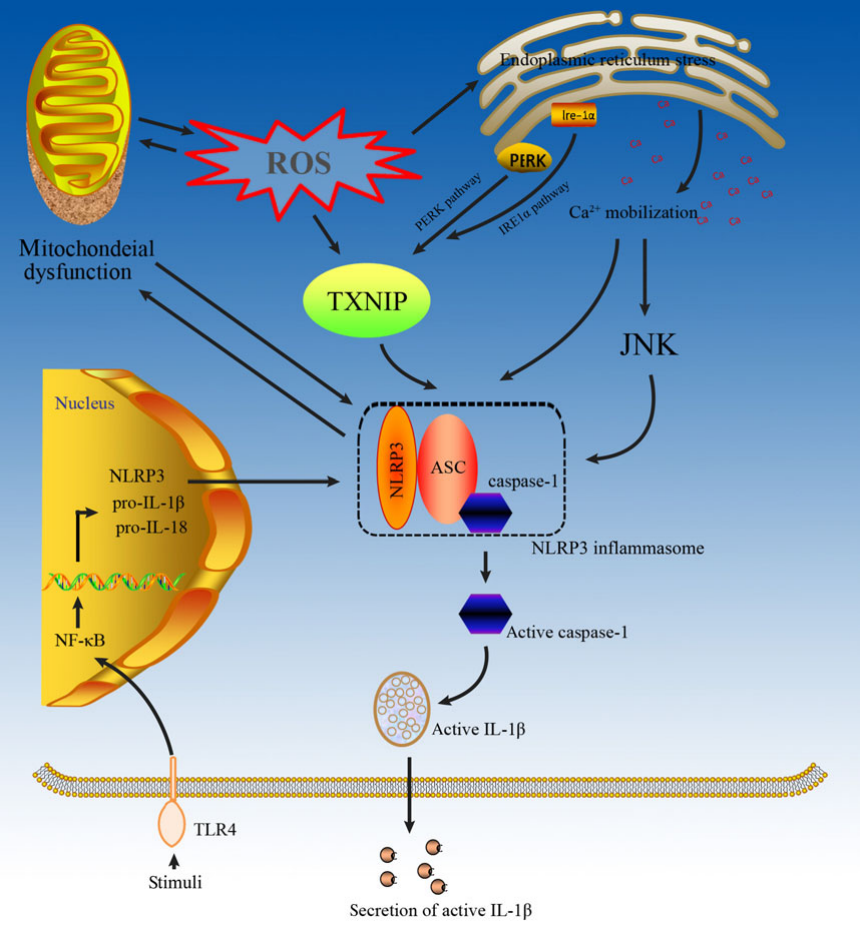
The role of the NLRP3 inflammasome in ORKD development. First, mitochondrial dysfunction activates the NLRP3 inflammasome directly by itself or indirectly by increasing ROS production. Second, ERS activates the NLRP3 inflammasome either in response to ROS or by inducing the production of TXNIP, which is released from TRX and binds to NLRP3 to induce NLRP3 inflammasome activation. ERS amplifies the NLRP3 inflammasome by enabling Ca^2+^ mobilization from the endoplasmic reticulum, which can be modulated *via* the C/EPB homologous protein. Furthermore, TLR4 and the Ca^2+^‐dependent MAPK‐JNK pathway may activate the NLRP3 inflammasome.

Mitochondrial dysfunction may occur prior to NLRP3 activation in the development of ORKD (Fig. 3), and mitochondrial dysfunction can directly activate the NLRP3 inflammasome 43 by itself or indirectly activate the NLRP3 inflammasome *via* ROS production 44. Meanwhile, NLRP3 proteins that are localized in mitochondria induce ROS *via* Smad independent of the inflammasome 55, which may amplify NLRP3 inflammasome activation. Moreover, mitochondria‐derived oxidative stress can induce mitochondrial dysfunction 56, which promotes further NLRP3 inflammasome activation. After being activated, the NLRP3 inflammasome may cause proteinuria‐induced renal tubular injury and promote the TGF‐β‐induced alteration of the proximal tubular cell phenotype to induce ORKD 57, 58.

NLRP3 inflammasome activation may cause mitochondrial damage in ORKD (Fig. 3) 59, suggesting that mitochondrial damage might be the consequence rather than the cause of inflammasomal activation 60. Moreover, ROS scavengers, such as N‐acetyl‐lysine (NAC), block the transcription of NLRP3 and pro‐IL‐1β but do not affect the direct activation of NLRP3 by classical NLRP3 agonists 61. Furthermore, NLRP3 deletion was shown to protect against renal injury *via* inhibiting mitochondrial dysfunction 58, suggesting that the NLRP3 inflammasome can induce mitochondrial dysfunction to exacerbate ORKD. Further studies are needed to understand how mitochondrial dysfunction and danger signals derived from damaged mitochondria affect NLRP3 inflammasome activation in ORKD.

Whether mitochondrial dysfunction induces NLRP3 inflammasome activation remains controversial. Sadatomi *et al*. showed that mitochondrial function was required for extracellular ATP‐induced NLRP3 inflammasome activation 62, and extracellular ATP has been shown to induce the loss of mitochondrial membrane potential and mitochondrial fragmentation in a manner different than that of other stimuli in primary mouse macrophages 63. Carbonyl cyanide m‐chlorophenyl hydrazone (CCCP), an uncoupler, and antimycin A, an inhibitor of the mitochondrial electron transport chain, could inhibit the release of IL‐1β induced by ATP but not by other stimuli 64. CCCP could not inhibit the ATP‐induced generation of ROS and cell death, both of which are known to promote IL‐1β release, but could inhibit the ATP‐induced activation of caspase‐1, a component of the NLRP3 inflammasome 64. These results suggest that mitochondrial function is required specifically for ATP‐induced NLRP3 inflammasome activation. In contrast to many previous reports that dysfunctional mitochondria promote NLRP3 inflammasome activation, functionally intact mitochondria appear to be required for NLRP3 inflammasome activation, depending on the stimulus 62.

## The NLRP3 inflammasome and endoplasmic reticulum stress

Endoplasmic reticulum stress plays a vital role in the development of CKD 65, 66, and inhibition of ERS has been shown to alleviate the progression of CKD 67, 68, 69. Similarly, recent studies have confirmed that ERS plays a specific role in the development of ORKD by inducing mitochondrial dysfunction 25, 70, and inhibition of ERS can effectively attenuate ORKD 70, 71. However, the mechanism by which ERS contributes to ORKD remains unclear.

The ERS/NLPR3 inflammasome axis may play a key role in the development of ORKD although few studies have reported their relationship in ORKD (Fig. 3). Li *et al*. found a significant overlap of NLRP3 with the ER marker calreticulin in renal proximal tubular cells, suggesting a crucial role of ERS in inflammasome activation 72. Furthermore, several studies have confirmed the existence of the ERS/TXNIP/NLRP3 inflammasome axis in multiple cells 73, 74, 75, 76. Increased ROS production through the NADPH oxidase pathway was shown to evoke ER stress *in vivo*, while enhanced unfolded protein response led to increased ROS production, exacerbating ER stress 77. TXNIP, originally characterized as a thioredoxin (TRX)‐binding protein that regulates the antioxidant function of TRX 78, is implicated in obesity and inflammation 79. In response to ROS production, ERS induces the production of TXNIP, which is released from TRX and binds to NLRP3 to induce NLRP3 inflammasome activation 80. The PERK and IRE1α pathways may mediate ERS‐induced TXNIP production 81, which has been shown to activate the NLRP3 inflammasome in the liver 82. In addition, blocking Ca^2+^ mobilization could inhibit the assembly and activation of the NLRP3 inflammasome complex 83. The homologous CCAAT/enhancer binding protein (C/EBP), a transcription factor that modulates the release of Ca2^+^ from endoplasmic reticulum, has been shown to amplify NLRP3 inflammasome activation, thus linking ERS to activation of the NLRP3 inflammasome. After being activated, the NLPR3 inflammasome not only promotes IL‐1β maturation following cleavage by caspase‐1, but also induces mitochondrial cell death 84.

## The NLRP3 inflammasome and inflammation

Inflammation plays a crucial role in the occurrence of ORKD, and rapid expansion of adipose tissue results in the aberrant production of pro‐inflammatory adipokines, leading to metaflammation, a state of low‐grade inflammation 2. Metaflammation, triggered by an increased metabolic rate resulting from excessive nutrient consumption 85, has been shown to facilitate the progression of obesity‐related diseases, especially renal disease 2. Adipocytes play a key role in metaflammation propagation, and several pathways, including the c‐junctional N‐terminal kinase (JNK) pathway and the Toll receptor pathway, have been shown to be activated in subjects with obesity and implicated in the induction of inflammation in metabolic tissues 86, 87. The pro‐inflammatory adipokines TNF‐α, IL‐6 and IL‐1β have been recognized as crucial mediators of adipose tissue inflammation 88, 89. These pathways and cytokines lead to inflammatory cell recruitment and activation in metabolic tissues, resulting in excessive release of inflammatory cytokines and in the development of ORKD.

The caspase‐1‐IL‐1β/IL‐18 axis is the basis of NLRP3 inflammasome‐induced inflammation in ORKD (Fig. 3), and release of the pro‐inflammatory cytokines IL‐1β and IL‐18 is controlled by the NLRP3 inflammasome 90. An animal study showed that the NLRP3 inflammasome‐caspase‐1‐IL‐1β/IL‐18 axis plays a vital role in ORKD 91. By regulating caspase‐1 activation, the NLRP3 inflammasome can promote IL‐1β and IL‐18 maturation 45. Moreover, IL‐18 can promote the production of TNF‐α, IL‐1β and inter‐cellular adhesion molecules *via* monocytes and macrophages, which can cause damage to the kidney 12. However, in an HFD mice model, activation of the NLRP3 inflammasome was associated with ASC, pro‐caspase 1, pro‐ IL‐1β and pro‐IL‐18 but not with mature IL‐1β and IL‐18 92. Chi *et al*. identified the NLPR3 inflammasome/IL‐23/IL‐17 axis in renal inflammation and renal fibrosis 93, which may cause ORKD. However, the role of NLRP3 in ORKD is not necessarily limited to canonical (caspase/IL‐1b/IL‐18–dependent) inflammasome activation 90 because NLRP3 has additional, non‐canonical roles in the development of ORKD *via* the TGF‐β1 signalling pathway 94, 95, caspase‐8 activation 96, and release of the pro‐inflammatory high‐mobility group box 1 97.

Multiple inflammatory pathways may coexist in ORKD, and several studies have found that TLR4 can activate the NLRP3 inflammasome 98, 99, 100 (Fig. 3). In hepatocytes, NLRP3 and TLR4 expression was up‐regulated after lipopolysaccharide stimulation 101. Moreover, CD36‐mediated TLR4/6‐IRAK4/1 signalling was shown to promote activation of the NLRP3 inflammasome in H9c2 cells 102. Furthermore, He *et al*. found that TLR4 mediated NLRP3 inflammasome activation in a mouse model of dextran sulphate sodium‐induced colitis 100. Taken together, these results suggest that the NLRP3 inflammasome activated by TLR4 is potentially the mechanism that leads to ORKD.

Interestingly, the Ca^2+^‐dependent MAPK‐JNK/NLRP3 inflammasome pathway may contribute to ORKD (Fig. 3). Chen *et al*. found that impairment of the Ca^2+^‐dependent MAPK‐JNK pathway suppressed NLRP3 inflammasome activation in bacteria 103 and demonstrated that JNK, a critical player in NLRP3 inflammasome activation, is a potential target of bacteria for manipulating inflammasomes. Moreover, activation of the MAPK‐JNK pathway is necessary for complete activation of the NLRP3 inflammasome through ASC 104.

Collectively, activation of the NLRP3 inflammasome through multiple pathways may play an essential role in the initiation of inflammation in ORKD. However, the crosstalk among these pathways is not well understood and requires further investigation.

## Conclusion and perspective

The aetiology and pathogenesis of ORKD are complicated. Oxidative stress, increased insulin and insulin resistance, and abnormal lipid metabolism are important risk factors of ORKD, but obesity and BMI are not. These ORKD risk factors appear to be closely related to the NLRP3 inflammasome, which is likely one of many mechanisms secondary to the structural and functional adaptions of nephrons to volume‐ and pressure‐related stress on the filtration barrier. Furthermore, the mechanism by which the NLRP3 inflammasome promotes ORKD development remains incompletely understood, and inhibition of the NLRP3 inflammasome not only suppresses the accumulation of renal cholesterol 105, but also ameliorates renal injury caused by obesity 91, 106. Therefore, targeting the NLRP3 inflammasome may provide a novel strategy for obesity‐induced kidney injury therapy.

## Conflicts of interest

All authors declare that there are no any ethical/legal conflicts involved associated with this article.
